# *Nosema ceranae* Escapes Fumagillin Control in Honey Bees

**DOI:** 10.1371/journal.ppat.1003185

**Published:** 2013-03-07

**Authors:** Wei-Fone Huang, Leellen F. Solter, Peter M. Yau, Brian S. Imai

**Affiliations:** 1 Illinois Natural History, Prairie Research Institute, University of Illinois, Champaign, Illinois, United States of America; 2 Roy J. Carver Biotechnology Center, Protein Sciences Immunological Resource Center, 307 Noyes Laboratory, Urbana, Illinois, United States of America; Stanford University, United States of America

## Abstract

Fumagillin is the only antibiotic approved for control of nosema disease in honey bees and has been extensively used in United States apiculture for more than 50 years for control of *Nosema apis*. It is toxic to mammals and must be applied seasonally and with caution to avoid residues in honey. Fumagillin degrades or is diluted in hives over the foraging season, exposing bees and the microsporidia to declining concentrations of the drug. We showed that spore production by *Nosema ceranae*, an emerging microsporidian pathogen in honey bees, increased in response to declining fumagillin concentrations, up to 100% higher than that of infected bees that have not been exposed to fumagillin. *N. apis* spore production was also higher, although not significantly so. Fumagillin inhibits the enzyme methionine aminopeptidase2 (MetAP2) in eukaryotic cells and interferes with protein modifications necessary for normal cell function. We sequenced the MetAP2 gene for apid *Nosema* species and determined that, although susceptibility to fumagillin differs among species, there are no apparent differences in fumagillin binding sites. Protein assays of uninfected bees showed that fumagillin altered structural and metabolic proteins in honey bee midgut tissues at concentrations that do not suppress microsporidia reproduction. The microsporidia, particularly *N. ceranae*, are apparently released from the suppressive effects of fumagillin at concentrations that continue to impact honey bee physiology. The current application protocol for fumagillin may exacerbate *N. ceranae* infection rather than suppress it.

## Author Summary

*Nosema ceranae*, a microsporidian pathogen described from Asian honey bees, was discovered infecting European honey bees coincident with early reports of Colony Collapse Disorder (CCD) and has been suggested to be a factor in honey bee declines. Use of fumagillin, the only known reliable treatment for the naturally occurring *Nosema apis*, subsequently increased but controversial field results were reported for *N. ceranae*. Fumagillin suppresses reproduction of microsporidia but disease prevalence and hive performance in treated apiaries were similar to untreated apiaries 6 months after treatment. We compared responses of *N. apis* and *N. ceranae* to the antibiotic at concentrations that simulated fumagillin degradation in hives during the honey bee foraging season when use of the drug is discontinued. *N. ceranae* spore production recovered at a higher fumagillin concentration than *N. apis*. At lower fumagillin concentrations, significantly more infective *N. ceranae* spores were produced in treated bees than in untreated infected bees. Protein profiles of bees fed fumagillin confirmed our hypothesis that fumagillin affects bee physiology at concentrations that no longer suppress *N. ceranae*. Use of fumagillin may increase the prevalence of *N. ceranae* and is potentially a factor in replacement of *N. apis* by *N. ceranae* in US apiaries.

## Introduction

Bicyclohexylammonium fumagillin, an antibiotic isolated from the fungus *Aspergillus fumigatus*, has been the only widely used treatment for nosemosis, or “nosema disease”, in western honey bees, *Apis mellifera*, [1], [2] for nearly 60 years [2]. The antibiotic (hereafter “fumagillin”), in the form of a 3% concentration for veterinary use, is considered to be the only effective treatment for *Nosema apis* infection and also suppresses the recently discovered microsporidian pathogen, *Nosema ceranae*, in honey bees [3].

*N. ceranae*, originally isolated from the Asiatic honey bee, *Apis cerana* [4] was discovered infecting *A. mellifera* in 2004 [5], increasing concerns about the impact of nosema disease on honey bee health. Microsporidia were correlated with declining populations of honey bees in the US [6], [7] and Spain [2]. Although fumagillin can control *N. ceranae* as well as *N. apis* at the manufacturer's recommended concentrations [3], several field studies have contradicted these results [8], [9] but no in-depth studies have been published. Since the discovery of *N. ceranae*, fumagillin sales have increased, and residues of the antibiotic were detected in harvested honey in the U.S. [10]. To reduce the residues, fumagillin treatment is prohibited during the foraging season [11], a time period that exceeds 6 months in most areas of the U.S. [12]. Hives are typically treated with the antibiotic once in the late fall and once in the early spring, usually prophylactically [13]. Fumagillin persists inside hives [2], and degrades over time [14].

The practice of periodic fumagillin treatment results in decreasing but nearly constant exposure of multiple generations of bees and pathogens to the drug. Although this practice appears to provide an environment conducive to selection of fumagillin-resistant *Nosema* strains, *N. apis* has evidently not developed resistance to the drug; however, studies have shown that *N. ceranae* can reestablish to pretreatment prevalence 6 months after treatments are terminated [2], [8]. Lower natural susceptibility to fumagillin or faster recovery from treatment could be a factor in the replacement of *N. apis* by *N. ceranae*, which apparently has occurred in North America and elsewhere [15], [16], [17].

Fumagillin inhibits the enzyme methionine aminopeptidase-2 (MetAP2) [18] and is known to block MetAP2 in *Encephalitozoon cuniculi*, a microsporidian pathogen of humans [19]. Most eukaryotes possess genes for two MetAP isoforms, MetAP1 and MetAP2, and apparently require either MetAP1 or MetAP2 to survive [18]. Microsporidia do not possess the MetAP1 gene [19], [20], making MetAP2 a logical target for suppression of microsporidian infection. The microsporidian MetAP2 gene, however, is homologous with MetAP2 genes in other eukaryotes, with approximately 60% similarity among all eukaryotic organisms [21]. Although fumagillin and analogous drugs are currently used for treatment of human microsporidiosis and certain cancers [22], fumagillin is known to be toxic to humans and other vertebrates by interacting with the MetAP2 enzyme, which is involved in protein maturation and post translation processes [23]. Honey bee queens and workers feeding on fumagillin have been shown to have significantly shorter lifespans [13], [24] but the potential toxicity of the antibiotic to honey bees when used to control nosemosis has received little study. Possible negative effects of fumagillin in insects, however, have been demonstrated in the greater wax moth, *Galleria mellonella* [25].

We fed low fumagillin concentrations to honey bees to evaluate effects of diminishing concentrations of the drug reported in bee hives [14] and documented increased production of *N. ceranae* spores and, to a lesser extent, *N. apis* spores in the treated bees. To determine if differences in susceptibility of *Nosema* spp. to fumagillin are reflected in MetAP2 sequences among apid species, and if honey bees are potentially susceptible to the drug, we compared MetAP2 sequences of the honey bee and the three described apid *Nosema* species, including *Nosema bombi*, a commonly observed pathogen of bumble bees, *Bombus* spp. [26], [27], [28]. *N. bombi* shares a close phylogenetic relationship with *N. apis* and *N. ceranae* but is not responsive to fumagillin treatment [29]. Based on MetAP2 sequence similarity and shorter lifespans of bees treated with fumagillin [13], [24], we hypothesized that fumagillin could also interact with the MetAP2 enzyme in honey bees. Computational comparison based on MetAP2 sequences of the pathogens and the honey bee is not yet optimal and there is no available *in vivo* enzyme dynamic comparison method; therefore, we performed 2D-gel electrophoresis (2DE) to evaluate the protein profiles in midgut tissues of uninfected honey bees fed concentrations of fumagillin corresponding to the bioassays of infected and treated bees. Our results suggest that declining levels of fumagillin in treated hives provide a window for hyperproliferation of microsporidia and that fumagillin continues to interfere with honey bee midgut physiology at levels that no longer suppress reproduction and maturation of *N. ceranae* and *N. apis*.

## Materials and Methods

### *Nosema* isolates

*Nosema apis* was provided by T. Webster at Kentucky State University and *N. ceranae* was isolated from honey bees from the University of Illinois at Urbana-Champaign apiary using methods identical with those used in previous studies [5]. *N. bombi* was isolated from *Bombus pensylvanicus* midgut tissues that were stored in liquid nitrogen as previously reported [28]. *N. apis* and *N. ceranae* were reproduced in caged bees, and mature spores were harvested from midgut tissues. Tissues were homogenized in glass tissue grinders, filtered through fine weave hardware mesh and centrifuged. Spore pellets were resuspended in sterile tap water and counted for immediate use in bioassays.

### Microsporidian spore production in honey bees treated with fumagillin

Brood frames from fumagillin-free colonies were held in growth chambers at 34.5°C, 65% relative humidity, 24 h dark. Newly emerged bees were transferred on a daily basis to cages consisting of 480 ml HDPE lidded plastic cups with tops cut out and screened with 3-mm hardware cloth [30]. The bees were fed with 50% sugar water (w/w), and pollen patties (15% pollen, Megabee) *ad libitum*. Five-day post-emergence adult bees were used for all bioassays; bees from four different hives that had not received fumagillin treatments for at least one year were used for trials conducted in 2011 and 2012. Bees were immobilized on ice, secured to a foam board with insect pins, and orally inoculated with 10^5^ spores of either *N. apis* or *N. ceranae* in 2 µl sugar water using a micropipetter. This dosage was selected to exceed the IC_100_ level of approximately 2×10^4^ spores (unpublished data for this *N. ceranae* isolate). Additional bees were randomly selected from the same brood frames and treated with sugar water without spores to verify that experimental bees had no background infection (negative control). Inoculated and negative control bees were transferred to new cages, 30 bees per cage per treatment, and held in growth chambers (30°C; 65% RH) after treatment. Beginning 24 h post inoculation until the experiment was terminated at 20 days post inoculation (dpi), inoculated bees were fed 50% sugar water *ad libitum* with selected concentrations of fumagillin. The tested concentrations included the manufacturer's recommended concentration of 25 mg/l or 1.0×, and 0.02, 0.01, 0.002, 0.001, 0.0002, 0.00006, 0.00001, and 0.0000033× the recommended concentration, and no fumagillin treatment as a positive control (Table 1). We focused on *N. ceranae*, currently the dominant microsporidian pathogen in US apiaries, but also conducted a limited number of tests of *N. apis*-infected bees (Table 1).

**Table 1:** Production of microsporidian spores in honey bees treated with varying concentrations of fumagillin.

Pathogen	Fumagillin concentrations	Mean spore count midgut tissues	Standard error	Total number of bees tested** (number of trials)
*Nosema apis*	no fumagillin (positive control)	3.07E+07	2.26E+06	70 (3)
	1.0×(25 mg/l)	NA*		45 (2)
	0.01×(250 µg/l)	1.71E+07^a^	2.50E+06	55 (3)
	0.002×(125 µg/l)	2.75E+07	2.94E+06	50 (2)
	0.001×(25 µg/l)	3.58E+07	3.40E+06	70 (3)
	0.0005×(12.5 µg/l)	3.29E+07	6.38E+06	25 (1)
	0.0002×(5 µg/l)	3.64E+07	3.00E+06	25 (1)
*Nosema ceranae*	no fumagillin (positive control)	2.70E+07	2.05E+06	120 (6)
	1.0×(25 mg/l)	NA*		45 (2)
	0.04×(1 mg/l)	3.3E+06^a^	5.37E+05	80 (3)
	0.02×(500 µg/l)	1.24E+07	1.36E+06	75 (3)
	0.01×(250 µg/l)	3.23E+07	2.88E+06	70 (3)
	0.002×(50 µg/l)	3.03E+07^b^	2.80E+06	80 (3)
	0.001×(25 µg/l)	5.28E+07^b^	5.32E+06	70 (3)
	0.0002×(5 µg/l)	3.68E+07^b^	5.60E+06	70 (3)
	0.000066×(1.6 µg/l)	2.86E+07^b^	1.92E+06	45 (2)
	0.00001×(0.25 µg/l)	2.87E+07^b^	2.08E+06	50 (2)
	0.0000033×(0.083 µg/l)	2.76E+07	2.28E+06	45 (2)

Midgut tissues of five bees were homogenized for each sample. Counts from day 10 to day 20 post inoculation were averaged.

* Spores observed were below the detectable number for the Petroff-Hauser counting chamber.

** Dead bees or fewer than five bees available at the last sampling were not counted.

a Significantly lower spore production than positive control (difference among trials was used as a cofactor).

b Significantly higher spore production than positive control (difference among trials was used as a cofactor).

Beginning 10 dpi for infected bees treated with 0.01, 0.001× fumagillin concentrations and untreated positive controls, five bees were randomly removed from each cage for evaluation, then five bees were sampled every 2 days until 20 dpi. Bees fed all other fumagillin concentrations were evaluated at 14 dpi based on the midpoint of peak spore production in the midgut tissues. Midgut and hindgut tissues were excised and separated before counting spores. Because caged bees seldom defecated inside the cages, the spore count in the hindgut was considered to be the accumulation of spores released from midgut cells for entire period of infection. The spores were isolated for counting by homogenizing the tissues of five bees per sample, midgut and hindgut separately, in a glass tissue grinder in 0.5 and 2 ml sterile distilled water, respectively, and were counted using a Petroff Hausser counting chamber under phase-contrast microscopy. Developmental stages of the spores, including primary (internally infective) spores, germinated primary spores, immature environmentally resistant spores (environmental spores) and mature infective environmental spores, were distinguished by refringence and morphological characters [31], and counted. Counts of mature spores were analyzed using one-way ANOVA (SPSS statistic software, IBM).

### DNA extraction and methionine aminopeptidase-2 sequencing and comparison

DNA was extracted from spores of *N. apis* and *N. bombi* using Chelex [28]. Each spore sample was mixed with Chelex buffer (5% Chelex, 5% Tween20, and 1 ng/ml proteinase K) and incubated in a thermocycler, 2 hr 56°C, 30 min 95°C. The samples were centrifuged at 13,000×g for 10 min and the supernatant containing DNA solution was used for amplification. The sequence of the *N. ceranae* MetAP2 gene was obtained from GenBank (acc. no. XM002996491). The degenerate primer set (NMetAP2F: GRG CDG CVG ARG CWC AYA G; NMetAP2R: TCR TCR CCT YTT GTW AGR AYY TC) was designed based on the alignment of MetAP2 genes of *N. ceranae* and *Encephalitozoon* spp. (GenBank acc. nos. AF440270, XM00307371, AY224694) and was used to amplify the MetAP2 gene from *N. apis* and *N. bombi*. Platinum taq (Invitrogen) was used for PCR following the manufacturer's suggested protocol with 3 nM (final concentration) of degenerate primers at annealing temperature 49°C. The DNA fragment was cloned into pGemT easy vector (Promega) and transferred into DH5α competent cells, and the DNA insertion was sequenced using vector primers. The sequences were identified and compared using BlastX, then aligned using ClustalX. The phylogenetic tree was analyzed by maximum likelihood using PhyML 3.0 [32] with settings suggested by ModelGenerator [33] with 1,000 times bootstrap.

### Protein isolation from fumagillin-treated bees and 2-D gel electrophoresis

Sugar water with fumagillin concentrations of 0.0, 1.0, 0.01 and 0.001× the manufacturer's recommended concentration were fed *ad libitum* to uninfected honey bees, 20 bees per cage. After 10 days of feeding, midgut sections of the alimentary tract were excised and cut vertically to remove the gut contents and peritrophic membranes. The tissues were homogenized in sterile phosphate buffered saline (PBS) and protein samples were prepared using Genotech Focus Total Proteome Kit with 1× protease inhibitor cocktail. The protein samples were processed using Genotech Perfect Focus dissolved in IPG rehydration buffer (GE) and loaded on IPG focusing strips (13 cm, pH 3–10, GE) for first dimension processing, and on BioRad Criterion 12% polyacrylamide gels in 1× MOPS buffer for the second dimension. The gels were stained in Sypro Ruby and scanned using a Typhoon 9400 multi-laser scanner. Major protein spots were excised from the 2DE gels and subjected to in-gel trypsin digestion and protein identification using ESI-LC/MS. Proteins were identified in Mascot (Matrix Science) and used standard protein BLAST to search NCBI-NR database specific for *Apis mellifera* proteins.

### Accession numbers/ID numbers for genes and proteins

Partial MetAP2 gene sequences of *Nosema* apis and *N. bombi* have been deposited in the GenBank database, accession nos. JQ927010 and JQ927011, respectively. The accession number of *N. ceranae* MetAP2 is XM002996491 [20] in GenBank. MetAP2 accession numbers for honey bee and human amino acid sequences are XP624161 and NP006829, respectively. The accession numbers of other MetAP2 sequences in analyses are AAC05144 (*Drosophila melangonster*), S45411 (*Saccharomyces cerevisiae*), AF440270 (*Encephalitozoon cuniculi*), AY224694 (*E. hellem*), AY224693 (*E. intestinalis*), AEI69245 (*Encephalitozoon sp.*).

## Results

### Bioassays

**Figure 1:**
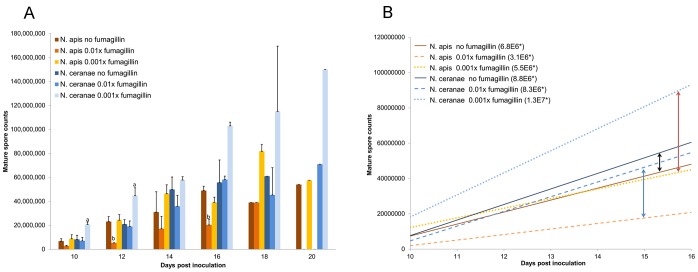
Mature *Nosema ceranae* and *Nosema apis* spores accumulated in hindgut contents of honey bee hosts. (A) Average total number of accumulated spores. Treatment protocols included positive controls (*N. apis* or *N. ceranae* infection, no fumagillin), and microsporidia plus 0.01× or 0.001× recommended concentrations of fumagillin. The recommended concentration suppressed spore production below the detection limit and is not included on the graph. One trial was conducted for the positive control 18 days post inoculation (dpi) and for all treatments 20 dpi; therefore, statistical evaluation was not performed for 18 and 20 dpi. Spore production significantly higher than the positive control is labeled “a”; spore production significantly lower than the positive control is labeled “b”. (B) Linear regression of spore accumulation in the hindgut contents, 10–16 dpi. Arrows indicate the difference between *N. ceranae* and *N. apis* within a treatment. *Slope represents daily spore accumulation.

Counts of *N. ceranae* spores in midgut tissues and hindgut contents varied significantly among fumagillin treatments and compared to untreated infected (positive control) bees. No spores were detected in bees inoculated with sugar water only (negative controls). Production of spores in midgut tissues reached a plateau phase with highest production capacity at 10 dpi and remained similar during the entire sampling period, 10–20 dpi, for both *N. ceranae* and *N. apis* infections (data not shown). The number of spores in the hindgut contents increased continually over the same period (Fig. 1A) but, after 16 dpi, spore counts were more irregular, perhaps the result of selecting surviving bees or those that defecated in the cages. We used 10–16 dpi data to generate a linear regression of spore accumulation in the hindgut (Fig. 1B). Slopes of regression lines for *N. ceranae* and *N. apis*, indications of daily spore production, were significantly different for all fumagillin concentrations. The difference between *N. ceranae* and *N. apis* is greater for honey bees treated with fumagillin, 165% and 129% higher for *N. ceranae* with 0.01× and 0.001× fumagillin, respectively. The slope was 30% higher for *N. ceranae* when bees were not treated with fumagillin. Unlike the 2011 cohorts, the 2012 test and negative control bees defecated in the cages and hindgut contents were lost, therefore spore counts were only made for midgut tissues in 2012.

**Figure 2:**
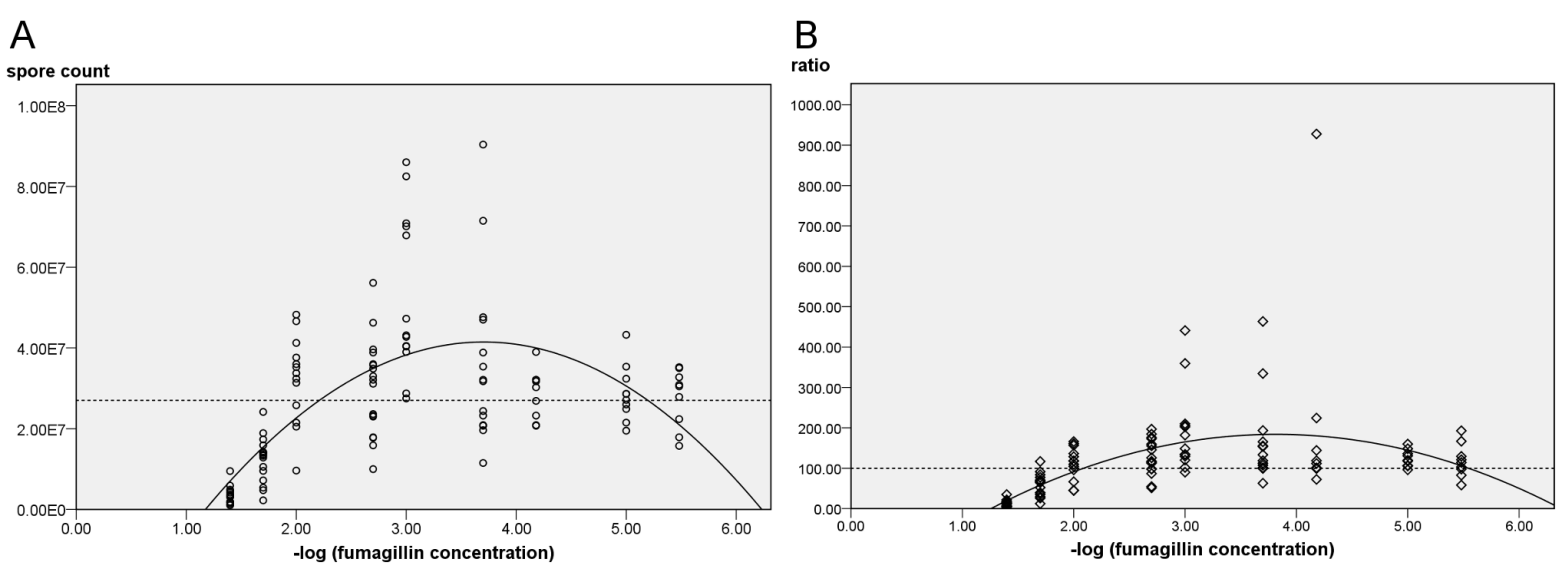
Midgut spore counts of *N. ceranae* inoculated bees treated with fumagillin. (A) Spore counts at descending fumagillin concentrations. Results from six trials were pooled. The regression matches the quadratic model (P<0.001), R^2^ = 0.418. (B) Normalized data setting the positive control at 100% for each trial (P<0.001; R^2^ = 0.272). Dashed lines represent midgut spore counts in infected, untreated bees.

The manufacturer's recommended concentration of fumagillin (1.0×) suppressed reproduction of both *Nosema* species, and the spore counts for midgut tissues and hindgut contents during the sampling period were below the resolution of the counting chamber. At a concentration of 0.04×, *N. ceranae* produced significantly fewer spores in the midgut tissues (P<0.001) than untreated controls; spore counts trended lower than the positive control at 0.02× (not significant; P = 0.086) but *N. ceranae* production began to recover at this concentration. At a concentration of 0.01×, *N. ceranae* produced a similar number of mature spores as positive control bees (P = 0.98 for midgut tissues; P = 1.00 for hindgut contents). In contrast, *N. apis* remained significantly suppressed at 0.01× (Table 1; P = 0.047 and 0.028 for midgut and hindgut, respectively). Both *Nosema* species were released from fumagillin suppression at 0.002× the recommended concentration. Significantly higher numbers of *N. ceranae* mature spores were produced in midgut tissues of infected bees treated with concentrations lower than 0.01× and higher than 0.0000033× the recommended fumagillin concentration than in positive control bees (P<0.05; Fig. 2). There was no significant difference in spore counts for *N. apis* infected bees treated with ≤0.002× fumagillin concentrations and positive controls. Although, the mean *N. apis* spore counts in hindgut contents trended higher (approximately 30% higher than in positive control bees), they were not statistically significant over three trials. With the exception of the recommended concentration, at all fumagillin concentrations tested, both *Nosema* species produced sufficient numbers of mature spores to infect numerous bees (Table 1). Hyperproliferation of *N. ceranae* was strongest at fumagillin concentrations between 0.001 and 0.0002×, then decreased with lower concentrations, although spore production was still significantly higher than positive controls at 0.00001 (1E^−5^)×(P<0.01). The lowest concentration tested, 0.0000033 (3.3E^−6^)×resulted in insignificant enhancement of spore production (P = 0.182).

Midgut spore counts corresponded with −log_10_ fumagillin concentrations (P<0.01) and the regression curve (Fig. 2A) predicts that the spore count of treated infected bees will equal the average counts of positive controls (no fumagillin) at 6.026 E^−6^×the recommended concentration. When data are normalized among trials to reflect the ratio between *N. ceranae*-infected positive control bees (no fumagillin) with treated infected bees, the predicted fumagillin concentration at which hyperproliferation no longer occurs is 2.239 E^−6^×(Fig. 2B).

### Comparison of MetAP2 genes

We investigated differences in the MetAP2 gene among the *Nosema* species that infect apid bees, *N. bombi*, *N. ceranae* and *N. apis*, and compared them to the honey bee and human MetAP2 genes. Degenerate primers amplified a partial coding domain sequence of the MetAP2 gene, 868 bp for *N. bombi* (GenBank acc. no. JQ927011) and 926 bp for *N. apis* (GenBank acc. no. JQ927010). These sequences lacked base pairs for 65 amino acids in the C-terminal and two amino acids in the N-terminal end that are reported for *N. ceranae* (GenBank acc. no. XM002996491) but included all fumagillin binding sites and metal ion coordinate sites necessary to evaluate the interaction between fumagillin and MetAP2. Similarity was 83% between *N. ceranae* and *N. bombi*, 73% between *N. ceranae* and *N. apis*, and 82% between *N. apis* and *N. bombi*.

**Figure 3:**
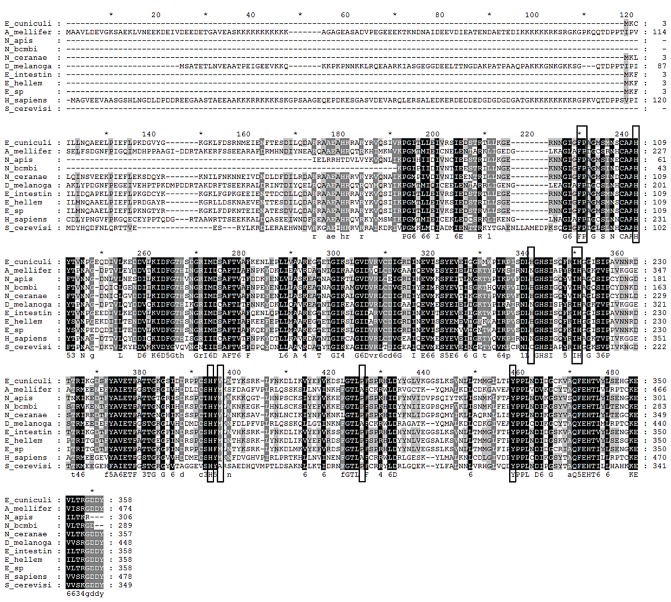
Alignment of translated MetAP2 genes. Circled amino acids are fumagillin binding sites; those marked with stars are metal ion binding sites. Species represented are *Encephalitozoon cuniculi, Apis mellifera, Nosema apis, Nosema bombi, Nosema ceranae, Drosophila melanogaster, Encephalitozoon intestinalis, Encephalitozoon hellem, Encephalitozoon* species, *Homo sapiens*, and *Saccharomyces cerevis*.

The MetAP2 genes from *Apis mellifera* (GenBank acc. no. XP624121), *N. ceranae*, *N. apis*, *N. bombi* and the mammalian microsporidium *Encephalitozoon cuniculi* (GenBank acc. no. AF440270), were translated using standard codec and aligned (Fig. 3). Binding site and coordinate site amino acid sequences were identical for honey bee and human MetAP2, and were identical among the microsporidia; *Nosema* spp. sequences differed from those of honey bees and humans at two fumagillin binding sites.

### Protein assays

**Figure 4:**
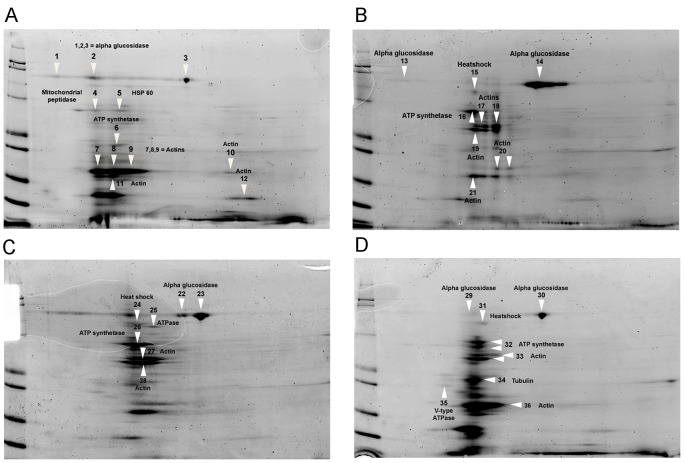
2-dimensional electrophoresis of midgut tissues of honey bees fed different concentrations of fumagillin. (A) 0.0×, no fumagillin (control). (B) 1.0× = manufacturer's recommended fumagillin concentration, 25 mg/L. (C) 0.01× the recommended concentration. (D) 0.001× the recommended concentration. Arrows indicate the proteins identified in Table 2. Disruptions of normal protein profiles corresponding to the fumagillin concentrations are observable.

We conducted protein assays (Fig. 4) to identify alterations in midgut proteins of uninfected honey bees caused by fumagillin treatment, including 1.0×, 0.01× and 0.001× the recommended concentration of the drug and no fumagillin treatment (control). Alterations in protein presence, quantity and position on 2DE gels were identified at all three fumagillin concentrations (Fig. 4). The ESI-LC/MS and Mascot (Matrix Science) library reliably identified 45 altered proteins related to energy metabolism, mitochondria, cellular structure and transport in the midgut tissues of honey bees (Fig. 4; Table 2). Alpha-actin protein isoforms were located in different positions on 2DE gels that correlated with fumagillin concentration and incremental changes were noted (Fig. 4). Another fumagillin concentration dependent protein was an isoform of alpha-glucosidase II; three isoforms were found in all treatments but with different ratios among the isoforms. These isoforms originate from the same gene in honey bee genome (Table 2). H+ transporting ATP synthase beta subunit isoform 1, located in mitochondria, appeared to have a different molecular weight for each fumagillin treatment, and a voltage-dependent anion-selective channel protein was found on the high PH value side of the gel in all fumagillin treatments (Fig. 4D) but not in the positive control bees.

**Table 2:** Proteins identified from infected midgut tissues in honey bees fed varying concentrations of fumagillin using 2D-gel electrophoresis (Figure 3).

Protein spot number	Identified protein	Protein description	Corresponded protein in honey bee genome
1, 2, 3, 13, 14, 22, 23, 29, 30	NP_001035326	alpha glucosidase 2 [*Apis mellifera*]	
4	XP_393509	PREDICTED: mitochondrial-processing peptidase subunit beta-like [*Apis mellifera*]	
5	XP_392899	PREDICTED: 60 kDa heat shock protein, mitochondrial-like [*Apis mellifera*]	
6	AAT06139	ATP synthase beta subunit [*Enallagma aspersum*]	PREDICTED: ATP synthase subunit beta, mitochondrial [*Apis mellifera*] (XP_624156)
7, 9	AAX51819	actin [*Melagraphia aethiops*]	actin related protein 1 [*Apis mellifera*] (NP_001172075)
8, 11, 17, 18, 19, 20, 21, 27, 28	XP_002128924	PREDICTED: similar to Actin, cytoplasmic [*Ciona intestinalis*]	actin related protein 1 [*Apis mellifera*] (NP_001172075.1)
10, 12	XP_002040521	GM18876 [*Drosophila sechellia*]	actin related protein 1 [*Apis mellifera*] (NP_001172075)
15, 24, 31	NP_001153524	heat shock protein cognate 3 [*Apis mellifera*]	
16	ABF18266	F0F1-type ATP synthase beta subunit [*Aedes aegypti*]	PREDICTED: ATP synthase subunit beta, mitochondrial [*Apis mellifera*] (XP_624156)
25	XP_623495	PREDICTED: v-type proton ATPase catalytic subunit A-like isoform 1 [*Apis mellifera*]	
26, 32	NP_001040450	H+ transporting ATP synthase beta subunit isoform 1 [*Bombyx mori*]	PREDICTED: ATP synthase subunit beta, mitochondrial [*Apis mellifera*] (XP_624156)
33	CAA28192	actin A3 [*Bombyx mori*]	actin related protein 1 [*Apis mellifera*] (NP_001172075)
34	NP_476772	alpha-Tubulin at 84B [*Drosophila melanogaster*]	tubulin alpha-1 chain-like [*Apis mellifera*] (XP_623220)
35	XP_001998980	GI24258 [*Drosophila mojavensis*]	PREDICTED: v-type proton ATPase 116 kDa subunit a isoform 1-like (XP_396263)
36	ABX46593	beta-actin [*Poecilia reticulata*]	actin related protein 1 [*Apis mellifera*] (NP_001172075)
37	XP_623725	PREDICTED: voltage-dependent anion-selective channel [*Apis mellifera*]	

## Discussion

Our laboratory bioassays corroborated field observations [2], [3] that fumagillin suppresses both *N. ceranae* and *N. apis* at the manufacturer's recommended concentration, but the two microsporidian species responded differently to decreasing fumagillin levels. At a concentration of 0.01× (250 µg/L) the recommended concentration, *N. ceranae* produced a similar number of mature spores in treated and untreated bees while *N. apis* remained suppressed. At a concentration of 0.001×, *N. ceranae* produced significantly more spores in the host midgut tissues (approximately 40%) than *N. apis* at the same treatment regime, and 24% more than in untreated *N. ceranae* infected bees. In addition, the average number of *N. ceranae* spores in the hindgut was approximately 80% higher than *N. apis* spores at 0.001× fumagillin concentration, and 150% higher than in the hindgut contents of untreated *N. ceranae-*infected bees. Spore production of both *Nosema* species increased at lower levels of fumagillin residue, although not significantly for *N. apis*, and *N. ceranae* spore production was double that of *N. apis*. We demonstrated that very low levels of fumagillin residue, possibly below the detection limit [14], affect the interaction between the microsporidia and the host. The higher number of *N. ceranae* spores produced in treated honey bees could potentially increase the pathological effects and transmission of the microsporidium but mortality did not significantly differ among fumagillin treatments of infected bees in our trials.

*N. apis* and *N. ceranae* produced similar numbers of mature spores in midgut tissues of untreated infected honey bees, results that corroborate those of Forsgren and Fries [34] and Paxton et al., [35]; however, the spore counts from hindgut contents of untreated bees were significantly higher for *N. ceranae* infections (approximately 45%) than for *N. apis* (P = 0.037). When results of mature spore counts in the midgut and hindgut are combined, *N. ceranae* produced significantly more spores than *N. apis* (P = 0.031), corresponding to results of Martin-Hernandez et al. [36]. Our results perhaps resolve some disparity in spore counts noted among laboratories [37].

Disparity between the midgut and hindgut spore counts was noted for *N. ceranae* in the fumagillin trials. The slope of the growth curve representing accumulation of spores in the hindgut from 10–16 dpi (Fig. 1B) indicates that mature spores are produced in the midgut of bees treated with 0.001× fumagillin faster than in untreated infected bees. The difference increases if 18–20 dpi data are included, but these data are based on one trial. Unfortunately, the honey bees we used in 2012 voided the hindgut contents during the treatment period and we could not continue this investigation. Nevertheless, the results suggest that a “snapshot count” of mature spores in midgut may not fully indicate the speed of *N. ceranae* proliferation, a phenomenon that has been also been suggested from tissue observations [38].

Field tests of apiaries in Spain reported the degradation of fumagillin to approximately 0.001× of the applied concentration 3 months after treatment termination [14]. Although the US Federal Drug Administration disallows fumagillin usage during the foraging season, marketable honey in U.S. was found to contain 60 ng/g of fumagillin residue [10], approximately 0.0024× the recommended treatment concentration. We calculated that fumagillin degradation in the field should be approximately −log10/month based on residues in the U.S. and field results from Spain. Using these calculations, the period during which hyperproliferation of *N. ceranae* occurs is approximately 2 to 5.5 months after cessation of fumagillin treatment. A more comprehensive field study, however, may be necessary to confirm these estimates.

Our results suggest that *N. ceranae* can resume spore production significantly earlier than *N. apis* and hyperproliferation of *N. ceranae* results in more than twice the spore production of *N. apis* at the same conditions. Without fumagillin, *N. ceranae* produces only slightly more spores than *N. apis* (Table 1; Fig. 1B) and has no other known significant competition advantage [37]. Less susceptibility to fumagillin and hyperproliferation in the presence of low residues of the drug, however, suggest a mechanism by which *N. ceranae* may outcompete *N. apis*. Although use of fumagillin apparently has not selected a resistant strain of *N. apis*, it may provide an advantage to the pathogen that possesses more natural resistance.

We looked for differences in the MetAP2 gene among apid microsporidia that might provide information about differences in susceptibility to fumagillin. The MetAP2 gene and amino acid sequences are most similar between *N. bombi* and *N. ceranae*, 83 and 80% respectively, but *N. apis* and *N. bombi* share a closer relationship in phylogenetic analysis of MetAP2 sequences. This suggests that MetAP2 is not obviously different among these *Nosema* species, yet *N. bombi* is apparently not affected by fumagillin treatment [29]. The relationship between MetAP2 phylogeny and sensitivity to fumagillin indicated that MetAP2 may not be the only factor influencing response to fumagillin, and different responses have been reported for other microsporidia and hosts [39], [40], [41]. Nevertheless, the high similarity of the honey bee MetAP2 suggested the possibility that honey bees are affected by fumagillin at concentrations at which *N. ceranae* hyperproliferates.

MetAP2 is known to be involved in various post translation modifications of multiple proteins [23], but the full extent of its action is not known. Protein analysis of midgut tissues of uninfected bees fed different concentrations of fumagillin confirmed our hypothesis that fumagillin interacts with host MetAP2 at concentrations that no longer suppress *Nosema* spp. At 0.001× the recommended treatment concentration, changes in structural and metabolic proteins were less dramatic than at higher concentrations but were still observable (Fig. 4). Although we did not identify specific proteins that, when altered, would allow hyperproduction of microsporidian spores, several proteins existing in isoforms, e.g. alpha-actin and glucosidase, were altered in all fumagillin treated groups. Alterations in actin proteins were shown to be necessary for successful infection by intracellular apicomplexan parasites [42], [43]. Alpha glucosidase II isoforms are necessary in carbohydrate metabolism [44], and other altered proteins (Table 2) are located in mitochondria. Nosemosis in honey bees causes additional energy stress [45], therefore changes in proteins involved in energy metabolism could influence the progress of infection. It is possible that the alteration of isoforms results from the interference of MetAP2 function in protein modification; most of the altered proteins we identified have universal effects on cell function.

Possible synergic effects between pesticides and *N. ceranae* were previously reported [46], [47], [48], and we found that effects of fumagillin were surprisingly similar and possibly stronger than the pesticides tested when it degraded to low levels. In contrast to pesticide exposure during honey bee foraging, fumagillin is applied to the hive directly by bee keepers to treat nosemosis. We have not investigated whether fumagillin usage has consequences for infection by other pathogens, but it is clear that the unintended consequence of its use could be exacerbation of *N. ceranae* pathogenesis. Fumagillin treatment is known to reduce microsporidian reproduction and is probably useful for protecting weak colonies [2], but the antibiotic may have unintended effects on the honey bee host, ultimately contributing to increased prevalence and pathogenicity of *N. ceranae*. Many variables could affect fumagillin concentration in hives post-treatment, including hive size, nectar flow and other factors. Current fumagillin application involves a treatment gap of 6 months or more and almost guarantees that the antibiotic will degrade to concentrations that allow release of the microsporidia and result in fast recovery of *N. ceranae* [2], [8]. In addition, the time period of *N. ceranae* hyperproliferation may reverse the benefits gained at the beginning of fumagillin treatment, resulting in indistinguishable performance between fumagillin treated and untreated hives [9]. Although field studies are necessary to determine if fumagillin use has value in specific situations, it is clear that new treatments for nosema disease are needed. Identification and development of drugs that will target the microsporidia without serious impacts on host physiology are critical for control of nosemosis.
